# More than a Thickener: Xanthan Gum as a Vehicle for the Herbicidal Extract of *Saussurea lappa* and Its Rheological Characterization

**DOI:** 10.3390/plants15020337

**Published:** 2026-01-22

**Authors:** Shafiu Mustapha, Bryan N. S. Pinto, Ângelo M. L. Denadai, Elson S. Alvarenga

**Affiliations:** 1 Department of Chemistry, Universidade Federal de Viçosa, Viçosa 36570-900, MG, Brazil; 2 Instituto Federal de Educação, Ciência e Tecnologia do Espírito Santo, Barra de São Francisco Campus, Barra de São Francisco 29800-000, ES, Brazil; 3 Pharmacy Department, Universidade Federal de Juiz de Fora, Governador Valadares Campus, Governador Valadares 35032-620, MG, Brazil

**Keywords:** xanthan gum, *Saussurea lappa*, rheological characterization, colloidal characterization, bioherbicide, phytotoxicity

## Abstract

The increasing demand for food is the driving force behind the search for novel, more selective, and less hazardous agrochemicals. Natural products are gaining prominence recently due to the promise of being green agrochemicals, but many natural products are poorly soluble in water, which reduces their applicability. In this work, we successfully formulated a water-insoluble *Saussurea lappa* root extract into a stable aqueous suspension using xanthan gum. The colloidal suspension was characterized by rheology, dynamic light scattering, and zeta potential. The results demonstrated that the suspension is a stable, sprayable, shear-thinning viscoelastic system (weak gel). A series of *S. lappa* solutions with xanthan gum were prepared and tested against five plant species, observing the inhibitory effect on the shoots and roots. The results were also compared with the commercial herbicide Dual. The *S. lappa* extract presented results comparable to or even greater than Dual for *Lactuca sativa*, *Cucumis sativus*, *Brachiaria decumbens*, and *Bidens pilosa*. However, it showed low inhibitory activity for *Sorghum bicolor*, highlighting its potential for selective weed control. This work illustrates xanthan gum as an effective vehicle for formulating insoluble natural products and demonstrates that *S. lappa* extract is a promising candidate for developing novel herbicides.

## 1. Introduction

The human population is steadily increasing. In 2022, the global population reached 8 billion; by 2030 it is expected to surpass 8.5 billion [1]; and by 2050 it is estimated to reach 10 billion people [2]. As the expansion of new arable lands is severely limited by environmental concerns, such as deforestation and preservation of biodiversity, a crucial approach to meet this growing demand for food [3] is to optimize the food productivity of the existing agricultural land [4]. One of the major challenges faced by modern agriculture is the prevalence of pests—such as weeds, bacteria, insects, and fungi [1]—which diminish crop productivity and directly impact the economy. Since the early 20th century, agrochemicals have been one of the many tools farmers have at their disposal to protect their crops. These tools address a critical economic issue, as crop pests and other diseases account for over USD 220 billion in losses globally each year [5]. Consequently, the agrochemicals market size in 2023 was USD 232.8 billion [6] and their application helps to prevent between 30 and 40% of annual global yield losses that would otherwise occur [7].

Despite these benefits, the use of conventional agrochemicals presents at least three major challenges that must be addressed. First, the pests and pathogens have developed resistance to many agrochemicals over the years [8,9,10]. Second, some agrochemicals are associated with issues such as environmental contamination and the presence of pesticide residues in food [3,11]. Third, the indiscriminate use of agrochemicals by the agricultural workers, often without following any safety protocols, exacerbates the problems of resistance and environmental contamination [12,13]. Therefore, it is crucial to develop novel agrochemicals that exhibit high efficacy at low doses, possess high selectivity, have minimal environmental persistence and impact, remain stable across a wide temperature range, and have low manufacturing cost. Equally important, they must be non-toxic to non-target organisms and have a novel mechanism of action to overcome existing resistance found in modern pests [14].

These limitations of conventional agrochemicals have driven researchers to search for novel agrochemicals, with nature as a rich source for chemical inspiration and diversity [15,16,17]. This can be attributed to natural products, which are biologically active compounds derived from animals, plants, marine organisms, and microorganisms [18,19]. Humans have been using natural products as therapeutic agents or as pesticides for centuries [20]. For instance, Sumerians used sulfur compounds to control insects and mice as early as 4500 years ago [21]. In recent years, organic chemists have been using the diverse molecular structure of many natural products as a template for the synthesis of novel agrochemicals with potent pesticidal and herbicidal activities [22,23]. Nonetheless, the use of natural products per se remains a relevant strategy in contemporary agriculture [24,25].

Among the diverse range of natural products, *Saussurea lappa*, a medicinal plant originating in the Himalayan region, emerges as a promising candidate. It typically grows at altitudes of 2500–3500 m [26] and is used in traditional medicine systems in countries including China, Pakistan, Japan, and India [27,28]. The plant has been known for over 2500 years [29] and it is known by many common names, for example, Costus (English), Kuth (English), Kust (Persian and Arab), Gostham (Tamil), Pachak (Hindi and Bengali), Postkhai (Kashmiri), and Koshta (Kannada) [30,31]. Morphologically, *S. lappa* is characterized as a tall perennial herb, typically reaching a height of 1–2 m. Its stem is upright, stout, and fibrous. A characteristic feature is its large taproot, which grows to about 60 cm and has a distinctive, strong odor. The plant’s leaves are notably large, approximately one meter long, with a lobed and stalked appearance. It has dark purple to blackish-blue flowers arranged in terminal heads. Its fruits have a cupped shape and are small (approximately 3 mm), hairy, and compressed [29,31,32]. The uses of *S. lappa* in traditional medicine are vast, from treating asthma and coughs to inflammation and ophthalmic conditions [30,33]. Since the 1950s, research has led to the isolation and identification of several natural products from *S. lappa.* These encompass a diverse range of compounds, with the most prominent of these being sesquiterpenes, alongside alkaloids, lignans, tannins, phenols, terpenes, and flavonoids [27,34,35]. Currently, the properties of natural products extracted from *S. lappa* are well known, such as antihypertensive, spasmolytic, immunomodulatory, anti-cancer, anti-inflammatory, antioxidant, and antimicrobial activities [35,36,37]. Despite its established applications in medicine, there is scope for further research into the application of *S. lappa* and its natural products as agrochemicals for crop protection.

Generally, natural products have limited solubility in water; hence, to use them as an agrochemical, it is necessary to solubilize them with an organic solvent [38,39]. However, many organic solvents are dangerous to human health and the environment due to their high volatility and environmental persistence [40,41]. The essential oil extracted from *S. lappa* is also poorly soluble in water, creating a challenge for its application. Therefore, developing strategies other than using conventional organic solvents is essential to overcome this solubility issue.

Xanthan gum, discovered by Allene Rosalind Jeanes in 1950 [42], is a nontoxic, bio-compatible, bio-degradable natural polysaccharide [43]. It is synthesized through the aerobic fermentation of sugars by bacteria of the genus *Xanthomonas* [44,45]. Physically, it is a dry, odorless, yellowish-white powder that is easily soluble in cold or hot water [43]. Due to its safety [6], xanthan gum is certified by the United States Food and Drug Administration and the European Union as a food additive [46]. Besides its well-known application in the food industry, xanthan gum is also used in drug delivery systems (tablets, films, hydrogels) [47], tissue engineering [42], cosmetic products [43], water treatment [48], soil improvement [49,50], concrete blends in the construction industry [51], and even in the petroleum industry [44]. Because of its qualities and lower production costs, the gum’s yearly global production is estimated to be 30,000 metric tons [52,53]. A key application of xanthan gum is as a dispersion stabilizer, a thickening agent, and a viscosity-increasing agent in aqueous solutions [44,45]. When the gum is dissolved in water with an insoluble substance (such as an oil extracted from *S. lappa*), it forms a three-dimensional network of polysaccharides, an aqueous gel, that increases the viscosity of the medium and traps the insoluble substance, preventing it from undergoing aggregation and sedimentation [6,43]. As a result, the mixture constitutes a stable colloidal suspension.

Thus, given the demand for new agrochemicals that are more selective and less harmful to the environment, the present work describes the evaluation of the potential herbicidal properties of a colloidal suspension of the oil extracted from the roots of *S. lappa* stabilized with xanthan gum. Furthermore, the physicochemical properties of this suspension, such as rheology, dynamic light scattering (DLS), and zeta potential (*ZP*), are also presented in this paper.

## 2. Results and Discussion

### 2.1. Dynamic Light Scattering and Zeta Potential

To assess xanthan gum as a stabilizing agent for the bioherbicide, suspensions containing different concentrations of *S. lappa* extract were prepared in a xanthan gum matrix. Colloidal characterization was performed after 20% (*v*/*v*) dilution in water, as the high viscosity of the undiluted samples prevents analysis by DLS and M3-PALS (Phase Analysis Light Scattering using Malvern’s 3rd-generation M3 technology). Furthermore, it is important to highlight that, if these samples are used commercially, they may be subjected to dilution during application.

**Figure 1:**
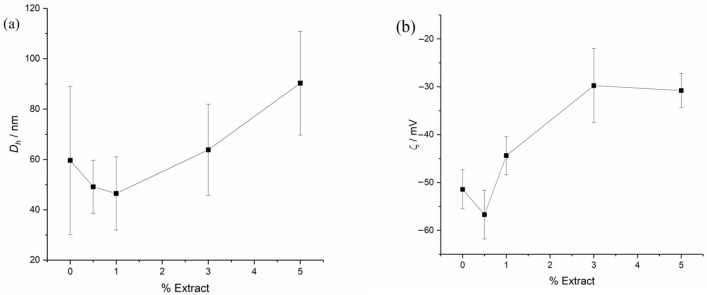
(**a**) *D_h_* (in nm) obtained by DLS (at 90°); and (**b**) *ZP* (in mV) data obtained by PALS (at 173° and an alternating voltage of 40 mV). Experiments performed at 25 °C.

Figure 1a,b show, respectively, the hydrodynamic (*D_h_*, in nm) and *ZP* (in mV) data of the xanthan solutions plotted against the extract concentration. As observed, both the size and the *ZP* generally increase with increasing extract concentration. As observed, there is a trend of size reduction to 1% extract, after which it increases. Furthermore, a slight increase in the zeta potential modulus is observed, which then starts to decrease.

Both data sets were subjected to a one-way ANOVA, followed by Tukey’s multiple comparison test at a significance level of 0.05 (the graphs with ANOVA results are available in the Supplementary Materials as Figures S4 and S5).

For the hydrodynamic diameter data, ANOVA revealed significant differences between the control (Xant 0.0%) and the samples at 0.5%, 1.0%, and 2.0% compared to Xant 5.0%, indicating that increasing the extract concentration tends to increase the hydrodynamic diameter. For the *ZP* data, the ANOVA showed significant differences among higher concentrations (Xant 3.0 and 5.0%) compared to the lower concentrations (Xant 0.0, 0.5 and 1.0%), also confirming the trend of change in *ZP* with increasing extract concentration.

The initial tendency of size reduction and the initial increase in the *ZP* modulus were attributed to ionic interactions between the extract components and the xanthan macromolecules. This interaction leads to disaggregation and the subsequent formation of smaller structures. However, the concomitant increase in size and reduction in the *ZP* modulus (i.e., *ZP* becoming less negative) were attributed to the partial neutralization of the aggregate’s electrical double layer by cationic components of the extract, thereby reducing repulsion and leading to an increase in aggregate size.

### 2.2. Rheological Characterization

The mechanical response of the gels under different stimuli (oscillation of amplitude, oscillation of frequency and heating) was investigated by its oscillatory behavior, in order to correlate the colloidal properties with macroscopic behavior. In oscillatory rheology, viscoelastic parameters named storage (*G*′), and loss (*G*″) moduli are viscoelastic properties that measure, respectively, the elastic component of the material (solid behavior) and the fluidity of the material (fluid behavior). Thus, when *G*″ is greater than *G*′, the material is liquid-like and when *G*′ is greater than *G*″, the material is solid-like. The resultant of these two parameters is named damping factor (*tan*(*δ*)), which is defined as the ratio: *tan*(*δ*) = *G*″/*G*′. Thus, *tan*(*δ*) < 1 indicates solid-like behavior, while *tan*(*δ*) > 1 signifies liquid-like behavior.

**Figure 2:**
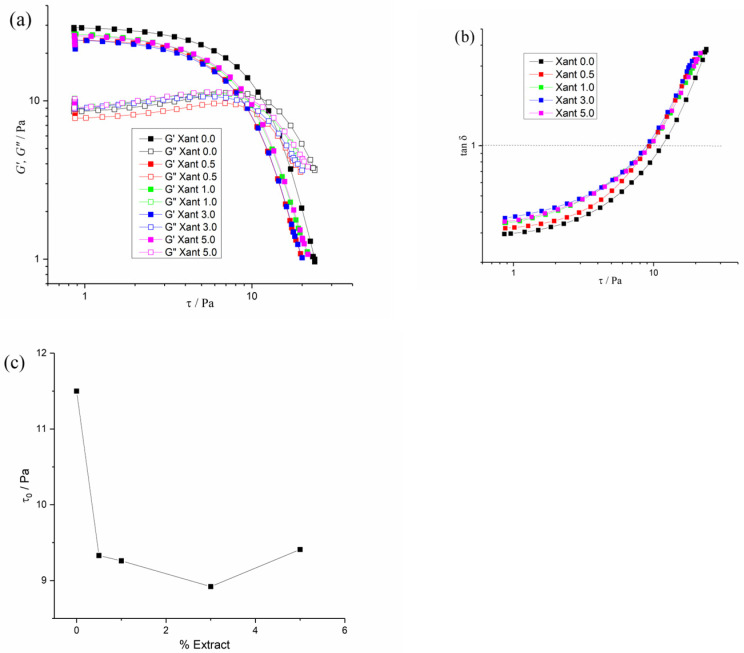
Strain amplitude oscillatory sweep, from 0.5% to 500% (in logarithm scale). (**a**) *G*′ and *G*″ Moduli plotted as a function of oscillatory shear stress (τ). (**b**) Damping factor as function of oscillatory shear stress (τ). (**c**) Yield stress values (τ_0_) against concentration of extracts, obtained from crossover of *G*′ and *G*″ in Figure 2a. Experiments performed at 87.92 rad/s (14 Hz) and 25 °C.

Figure 2a illustrates the viscoelastic behaviors for the Xant 0.0 to 5.0% of extract. Independent of the structuration degree, all fluids exhibited a reduction in *G*′ and *G*″ with the deformation. This behavior is classified as “strain thinning” viscoelastic fluids (analogous to pseudoplasticity in steady shear), according to Hyun et al. [54]. For these materials, polymer chains are in a state of entanglement in the small strain region, where *G*′ and *G*″ are constant. As the strain is increased, polymer chains disentangle, and then align with the flow field, leading to reduction in the *G*′ and *G*″ modulus [55]. Notably, strong intermolecular interactions were observed for all materials, as *G*′ remained higher than *G*″ over a wide range of deformation.

Moreover, a crossover between *G*′ and *G*″ was observed for all samples. This crossover is attributed to the existence of long-range interactions, which is responsible to create a yield stress (τ_0_). This crossover can be ascribed to a solid–liquid-like transition induced by a mechanical stress. It is hypothesized that in this transition, the external force breaks down specific elastic domains, causing fluidity. Beyond τ_0_, rupture of the intermolecular tridimensional structure of the fluid is expected, with similar solid-fluid transition [56,57]. In Figure 2a, the yield stress can be observed at *tan*(*δ*) = 1, when *G*′ = *G*″.

However, the magnitude of τ_0_ varied with the extract concentration, as shown in Figure 2c. As observed, there is a strong decreasing trend in τ_0_ values with the extract concentration, due to the reduction in the size of aggregates, caused by interactions of extract components with the macromolecule. The magnitude of *tan*(*δ*) also follows the same tendency. The slight increase in rigidity observed for the Xant 5.0% sample may be due to the increase in the size of the aggregates, combined with the increase in solid content.

**Figure 3:**
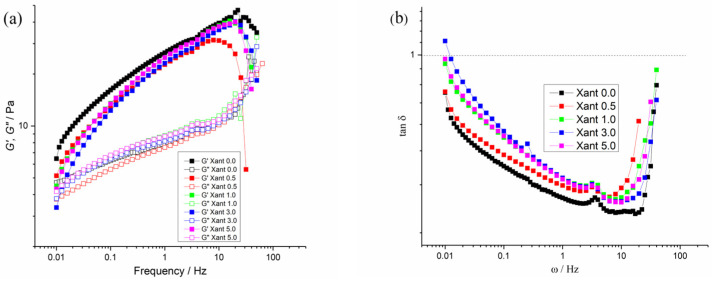
Frequency oscillatory sweep, from 0.0 to 628 rad/s (0.0 to 100 Hz, in logarithm scale). (**a**) *G*′ and *G*″ Moduli plotted as a function of frequency of oscillation (*ω*/Hz). (**b**) Damping factor as a function of oscillatory shear stress (*ω*/Hz). Experiments were performed at 87.92 rad/s (14 Hz) and 25 °C.

The frequency sweep experiments are shown in Figure 3, where both *G*′ and *G*″ moduli and *tan*(*δ*) are plotted against oscillation frequency [58]. For all samples, *G*′ > *G*″ in the overall range of frequency, following the same trend observed in the amplitude oscillation experiment. The dependence of viscoelastic properties on frequency is better observed in the *tan*(*δ*) graph in Figure 3b, where the system exhibits a tendency towards structuration with increasing frequency.

This behavior is typical of low-concentration macromolecular solutions (viscoelastic liquids), showing an apparent fluid character with a tendency towards a crossover in the high-frequency regime. They behave as viscous liquids at low frequency and as an elastic damper at high frequency [59].

As observed, the Xant 0.5% sample exhibited a distinct anomaly at high frequencies, characterized by a sharp deviation near 10 Hz. While the precise mechanism is complex, we hypothesize that this phenomenon arises from ionic interactions between the extract components and the xanthan macromolecules. As indicated by the DLS and *ZP* results, these interactions lead to disaggregation and the formation of smaller structures, which appear to be weaker and more susceptible to disruption under high-frequency oscillatory shear.

Regarding the damping factor, Figure 3b reveals *tan*(*δ*) peaks located at 3–4 Hz across all systems, apparently without any influence from extract concentration. In oscillatory rheology, such peaks observed, particularly in damping factor graphs, can be attributed to the relaxation (or partial relaxation, depending on their intensity) of the internal network, when the material exhibits greater energy dissipation (liquid-like behavior) induced by the external stimulus—in this case, the frequency sweep shearing. These phenomena consequently lead to changes in the aggregation state of the system. Since the peaks occur at the same frequency values for all systems, it can be inferred that the extract concentration does not influence this phenomenon and that it represents an intrinsic property of the free fraction of the macromolecules, which do not interact with the extract components.

**Figure 4:**
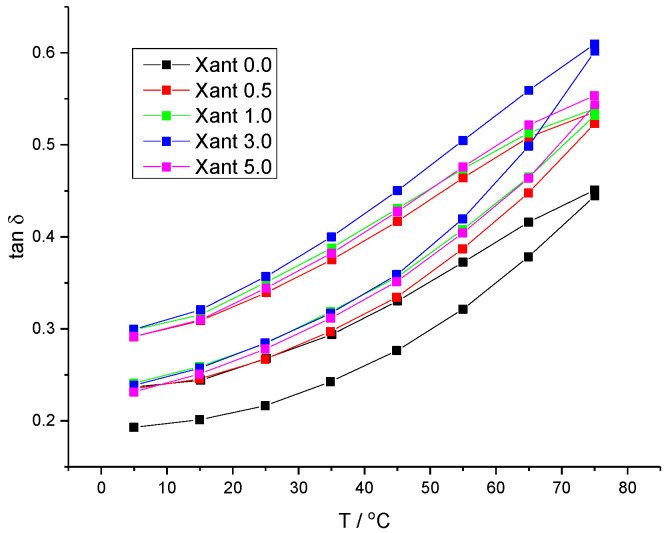
Temperature dependence of *tan*(*δ*) on heating of gels. Experiments were performed at *γ* = 0.05%, *ω* = 1 Hz and 5 °C/min.

Thermal behavior was also assessed via temperature ramp experiments (Figure 4). All xanthan solutions exhibited *tan*(*δ*) < 1 over the entire temperature range, consistent with the behavior of solid-like materials. However, a gradual increase in *tan*(*δ*) upon heating indicates a shift toward more liquid-like behavior, attributed to the increased kinetic energy of the polymer and solvent molecules.

Furthermore, pronounced thermal hysteresis was observed; the heating and cooling curves did not superimpose. Since the cooling curves exhibit *tan*(*δ*) values lower than those of the heating curves, a more rigid character was observed upon cooling. This behavior was attributed to the loss of water molecules via evaporation during the heating cycle.

**Figure 5:**
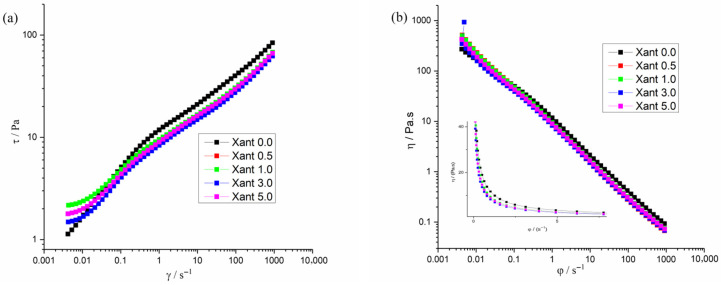
(**a**) Flow and (**b**) viscosity curves with ramp of shearing from 0.001 to 1000 s^−1^, at 25 °C.

Figure 5 presents the steady flow experiments, which have been used in order to simulate the behavior of the sample under stationary flow, where the shear is able to disrupt the overall microstructure of the material. As can be observed, the samples showed strong pseudoplastic behavior, with drastic decrease in viscosity and increasing shear rate. This reduction in viscosity is usually attributed to the alignment of the macromolecules or nanostructures [60,61] to the flow field imposed by the rotor [61]. Moreover, it can be observed that, above 5 s^−1^ (see insert in Figure 5), the samples reach the Newtonian regime, with viscosity remaining constant independent of the shear rate.

These data show that upon stationary flow, the samples are practically indistinguishable. The overall disruption of the microstructure suggests that the interactions involving the extract are not sufficiently strong to resist intense shear flow.

### 2.3. Biological Assay

Sesquiterpene lactones have been extracted, isolated, and characterized from *S. lappa.* These compounds have been used as templates for promising herbicides. In a recent study, dehydrocostuslactone (DHC, Figure S1) was isolated, and seven novel epoxy derivatives were synthesized, which demonstrated significant inhibitory activity against three plant species (*Allium cepa*, *Lepidium sativum*, and *Lactuca sativa*) [34]. Costunolide (Figure S2), another natural product recently isolated from *S. lappa*, was modified via a microwave-assisted Cope reaction, catalyzed by palladium (II), yielding five new derivatives. Three of these molecules were tested against etiolated wheat (*Triticum aestivum*) coleoptiles to assess their biological potential, and the results demonstrated significant phytotoxicity [62]. These two compounds, DHC and costunolide, are examples of the potential *S. lappa* holds as a source for novel agrochemicals.

The phytotoxic potential of the *S. lappa* root extract was evaluated using five selected plant species. *Lactuca sativa* (lettuce) and *Cucumis sativus* (cucumber) were selected as model organisms due to their high sensitivity and established use in standard phytotoxicity protocols [63,64]. To explore the extract’s potential as a bioherbicide, *Bidens pilosa* and *Brachiaria decumbens* were included as examples of dicotyledonous and monocotyledonous weeds, respectively, due to their widespread prevalence and impact on agriculture [65,66,67]. Finally, *Sorghum bicolor* was included to verify phytotoxicity on a standard grain crop [68]. This diverse selection allows for the study of both broad-spectrum activity and selectivity between monocots and dicots.

While the biological assays in this study utilized direct application to validate phytotoxicity, the rheological data serve as a predictive tool for future field application. The shear-thinning behavior observed indicates that the formulation, although viscous at rest to prevent sedimentation, will structurally break down under the high shear of a spray nozzle, allowing for uniform atomization in practical agricultural scenarios.

The results of the bioassay are presented in tables (Supplementary Materials) and bar graphs with their respective error bars (representing ± standard error). Positive values indicate stimulation of growth, while negative values indicate inhibition of growth for the shoots and roots of the tested plant species. The biological assay results are presented as the Relative Growth Change (RGC). All inhibition or stimulation percentages were calculated relative to the vehicle control (xanthan gum + DMSO), ensuring that the reported effects are attributable solely to the *S. lappa* extract.

#### 2.3.1. *Lactuca sativa*

**Figure 6:**
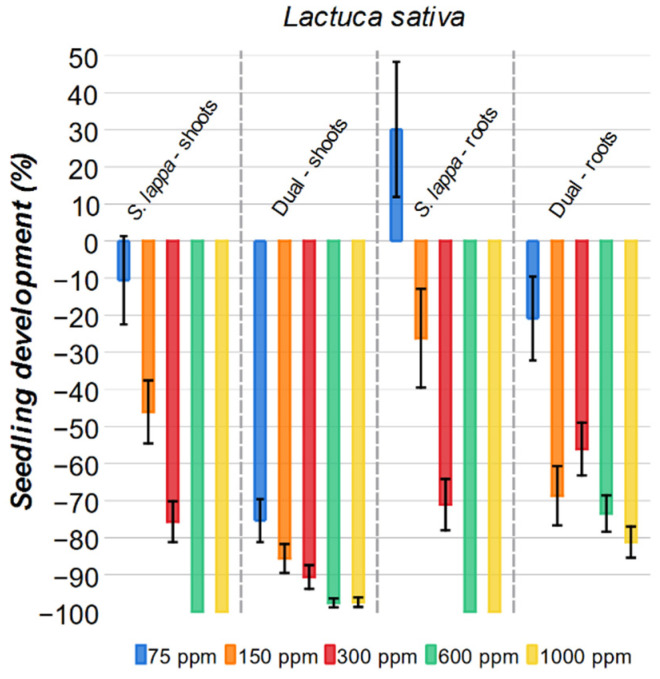
Effect of *S. lappa* extract and the commercial herbicide Dual on *Lactuca sativa* shoots and roots development. The error bars represent the standard error.

The *S. lappa* extract presents a significant impact on the growth of *Lactuca sativa* (Figure 6). For the aerial parts, all *S. lappa* solutions diminished the growth of the plant. At 75 ppm, a relatively minor inhibition was observed, 11%, but with the solution at 300 ppm, a highly significant 76% inhibition was obtained. The 600 ppm and 1000 ppm solutions were comparable to the commercial herbicide Dual, with both achieving a 100% inhibition rate at the same concentration as the commercial herbicide.

For the roots of lettuce, a biphasic effect was observed. The 75 ppm solution of *S. lappa* stimulates root growth by 30%, but at all higher concentrations, only inhibition was observed. Notably, when compared with Dual, the *S. lappa* extract at 300 ppm, 600 ppm, and 1000 ppm shows a greater inhibitory (15–25%) effect on the roots than Dual.

#### 2.3.2. *Brachiaria decumbens*

**Figure 7:**
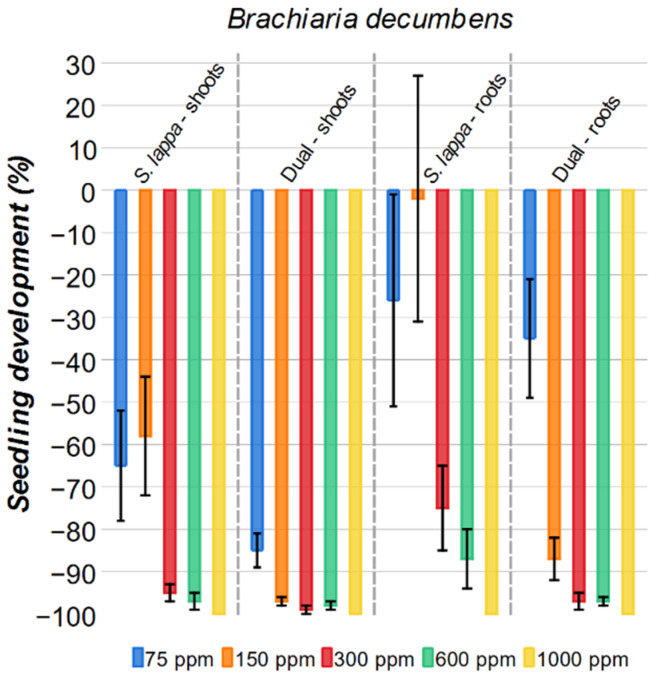
Effect of *S. lappa* extract and the commercial herbicide Dual on *Brachiaria decumbens* shoots and roots development. The error bars represent the standard error.

When evaluating the results (Figure 7), brachiaria grass was also sensitive to the *S. lappa* extract, showing growth inhibition at all tested concentrations. For the aerial parts, the *S. lappa* solution at 75 ppm and 150 ppm presented a 65% and 58% inhibition rate, which is outperformed by the commercial herbicide Dual. However, at higher concentrations, 300 ppm, 600 ppm, and 1000 ppm, the *S. lappa* extract achieved results comparable to Dual, with 95%, 97%, and 100%, respectively. A comparable trend in the inhibition rate was observed for the roots of *Brachiaria decumbens*; however, a distinct threshold for biological activity was noted. While the *S. lappa* solutions at concentrations of 300 ppm, 600 ppm, and 1000 ppm presented significant inhibitions (75%, 87%, and 100%, respectively), lower concentrations of 75 ppm and 150 ppm showed high variability and reduced efficacy. Weeds typically exhibit high variability in biological assays because their seeds are collected in the field without prior selection and testing, unlike crop seeds. It should be noted that the results obtained for crop species show only minor variations, since their seeds are pre-selected and tested, exhibiting germination rates higher than 99% (under controlled conditions). It is also noteworthy that at higher extract concentrations, the deviations are small. For the roots, the 1000 ppm concentration produced a response similar to that of the commercial herbicide.

#### 2.3.3. *Cucumis sativus*

**Figure 8:**
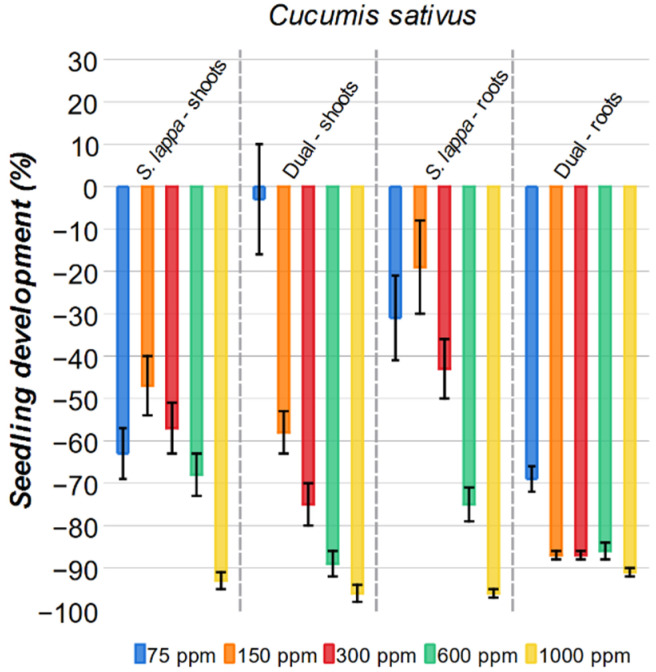
Effect of *S. lappa* extract and the commercial herbicide Dual on *Cucumis sativus* shoots and roots development. The error bars represent the standard error.

Similarly to *Brachiaria decumbens*, *S. lappa extract* was effective at inhibiting *Cucumis sativus*, but to a lesser extent (Figure 8). For the shoots, all concentrations of *S. lappa* solutions showed an inhibitory effect, ranging from 47% at 75 ppm to 93% at 1000 ppm. However, only the 1000 ppm solution delivered inhibition comparable to the commercial herbicide (96%). A similar pattern was also observed for the roots of cucumber. A 31% and 19% inhibition rate is observed for the 75 ppm and 150 ppm of *S. lappa*, and when concentrations are increased to 300 ppm and 600 ppm, inhibition also increased to 43% and 75%, respectively. Notably, for the 1000 ppm *S. lappa* solution, the inhibition for the roots was 96%, slightly more effective than the commercial herbicide Dual (93% inhibition) at the same concentration.

#### 2.3.4. *Bidens pilosa*

**Figure 9:**
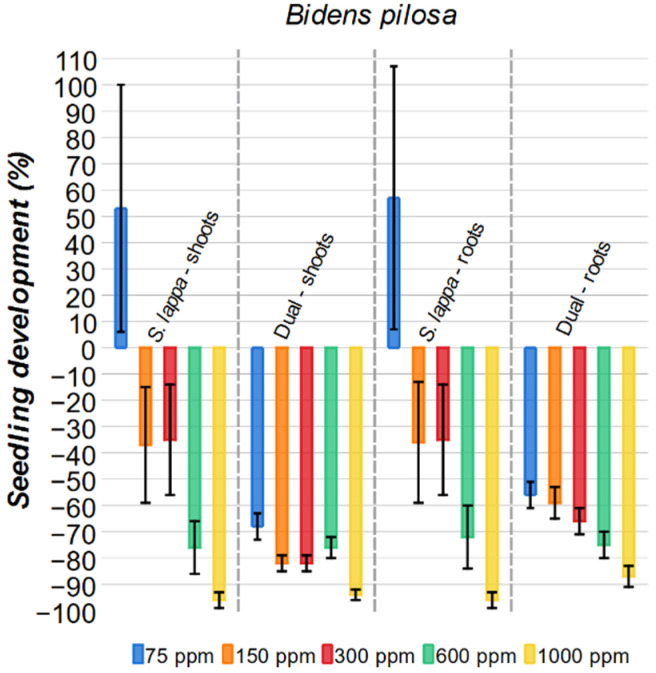
Effect of *S. lappa* extract and the commercial herbicide Dual on *Bidens pilosa* shoots and roots development. The error bars represent the standard error.

For *Bidens pilosa* tests, a biphasic effect was observed as well (Figure 9). The 75 ppm *S. lappa* solution demonstrated a stimulatory effect on the shoots, with a 53% growth. At higher concentrations, 150 ppm and 300 ppm, the extract had a modest 37% and 35% inhibition, respectively. These values are considerably lower when compared to the same concentration of the commercial herbicide Dual, with an 82% inhibition rate at both concentrations. On the other hand, the highest concentrations for the *S. lappa*, 600 ppm and 1000 ppm, showed a substantial inhibition (76% and 96%, respectively), values that are comparable to those obtained by Dual.

A similar biphasic effect was also present for the root of *Bidens pilosa*, with the 75 ppm solution stimulating root growth by 51%. As with the shoots, increasing concentration led to a more robust inhibitory effect on the roots. The commercial herbicide was more effective at 150 ppm and 300 ppm (59% and 66% inhibition, respectively) than the *S. lappa* solutions of the same concentration (36% and 35%, respectively). The 600 ppm *S. lappa* solution had a similar inhibition result (72%) to Dual (75%). However, the 1000 ppm solution (96%) was more inhibitory than the commercial herbicide (87%).

#### 2.3.5. *Sorghum bicolor*

**Figure 10:**
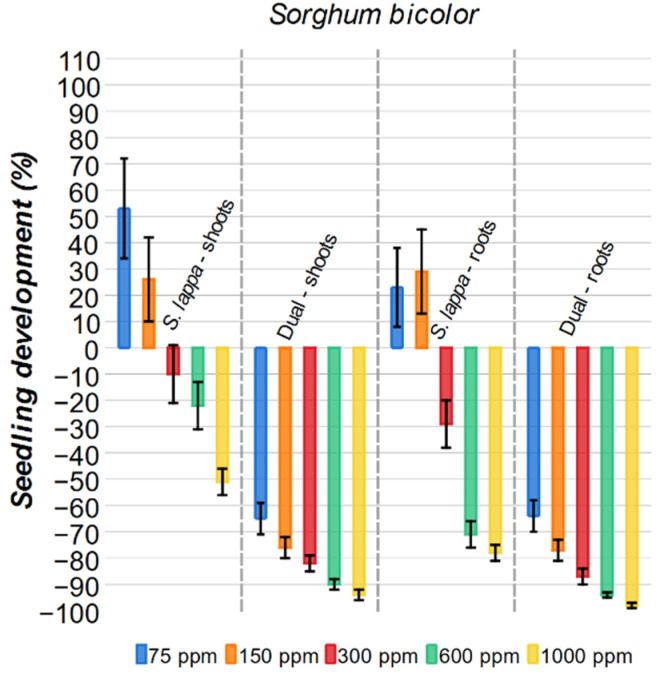
Effect of *S. lappa* extract and the commercial herbicide Dual on *Sorghum bicolor* shoots and roots development. The error bars represent the standard error.

The aerial parts of *Sorghum bicolor* demonstrated a higher resistance to the *S. lappa* extract compared to the other plants tested in this work (Figure 10). The *S. lappa* solutions of 75 ppm and 150 ppm presented a growth stimuli (53% and 26%, respectively) for the aerial parts of *Sorghum bicolor*, in contrast with the inhibitory effect of the commercial herbicide. Dual at the same concentrations (65% and 76%, respectively). The other *S. lappa* solutions, 300 ppm, 600 ppm, and 1000 ppm, were more inhibitory than the previous ones, but at modest levels (10%, 22%, and 51%, respectively). Overall, the Dual herbicide was significantly more effective at inhibiting the aerial parts of *Sorghum bicolor* at all concentrations.

A similar trend was observed for the roots. The 75 ppm and 150 ppm of the extract stimulated the roots, with 23% and 29%, respectively, while the commercial herbicide, at the same concentration, inhibited the growth with a rate of 64% and 77%, respectively. At higher concentrations (300 ppm, 600 ppm, and 1000 ppm), the extract’s inhibitory effects on the roots were stronger (29%, 71%, and 78%, respectively) than those observed for the aerial parts. Nonetheless, these results were still lower than those achieved by Dual at the same concentrations (87%, 94%, and 98%, respectively).

In summary, the biological assays reveal that the *S. lappa* extract stabilized with xanthan gum has a high phytotoxic activity. Its results are comparable to the commercial herbicide, Dual, against broadleaf species (*L. sativa*, *C. sativus*, *B. pilosa*) and the monocot weed, *B. decumbens*, at higher concentrations. Nonetheless, we observed a different behavior for *S. bicolor*. While Dual caused severe inhibition to *S. bicolor*, the extract showed significantly lower toxicity to this crop. This contrast highlights the *S. lappa* extract’s potential as a selective bioherbicide because it had a high efficacy against target weeds but a reduced toxicity to *S. bicolor*.

## 3. Materials and Methods

### 3.1. Extraction

The powdered roots of *Saussurea lappa* was imported from China (purchased commercially via Alibaba.com, Hangzhou, China, on 11 May 2011). A total of 500 g of the powdered material was extracted in five batches of 100 g each. Each batch was wrapped in a thimble and placed in a Soxhlet extractor. Extraction was performed with ethanol (Sigma-Aldrich, St. Louis, MO, USA) at 75–80 °C for 8 h, until the solvent in the siphon became colorless. The solvent was removed under reduced pressure using a rotary evaporator. Subsequently, a liquid–liquid extraction was carried out on the crude extract using a mixture of water and dichloromethane (1:1, *v*/*v*). The aqueous phase was separated from the organic phase using a separatory funnel. Anhydrous MgSO_4_ was added to the organic phase to remove traces of water, followed by filtration. Dichloromethane was removed under reduced pressure using a rotary evaporator. Finally, for the bioassays, the crude extract was dissolved in 0.3% (*v*/*v*) DMSO (Sigma-Aldrich, St. Louis, MO, USA) and stabilized in a 0.5% (*m*/*v*) xanthan gum solution. However, different concentration ranges were used in the biological experiments and in the physicochemical analyses. In the rheological experiments, higher concentrations of both polymer and extract were employed, whereas in the biological assays, very low concentrations (ppm range) were used. It is important to emphasize that, during the preparation of formulations for environmental applications—including herbicidal formulations—concentrated solutions or dispersions are typically produced and subsequently diluted in situ during application. This approach is commonly adopted to reduce volume, thereby facilitating transportation and commercialization.

### 3.2. Preparation of Colloidal Solutions for Physicochemical Studies

Xanthan gum formulations containing different extract concentrations were prepared while maintaining a constant DMSO content. A 2% (*m*/*v*) xanthan gum solution was prepared and allowed to fully hydrate. Four extract concentrations (0.5%, 1%, 3%, and 5% *m*/*v*), relative to the 2% xanthan gum base, were formulated. Briefly, 50, 100, 300, and 500 mg of the extract were individually dissolved in 0.5 mL of dimethyl sulfoxide (DMSO) in separate 10 mL glass vials. Subsequently, volume was adjusted to 10 mL with the pre-prepared 2% xanthan gum solution, and the mixture was stirred until homogeneous. A blank formulation was prepared by adding 0.5 mL of DMSO to a 10 mL vial and diluting to volume with the 2% xanthan gum solution. All formulations had identical final volumes, xanthan gum concentrations, and DMSO contents. These formulations were used for the rheology characterization, but due to the high viscosity of the formulations, all samples were diluted to 20% (*v*/*v*) in Milli-Q^®^ (Sigma-Aldrich, St. Louis, MO, USA) water prior to the DLS and m3-PALS analyses.

### 3.3. Dynamic Light Scattering and Zeta Potential Measurements

A Malvern Zetasizer Nano series instrument (Nano ZS90, Malvern Instruments, Malvern, UK) was used to measure *D_h_* and *ZP* of the nanostructures present in the xanthan extract solutions. Particle size was determined by *DLS* using monochromatic light (10 mW He–Ne laser, 632.8 nm wavelength), with scattering intensity measured at a 90° angle. The suspensions were placed in Malvern standard polyethylene cuvettes with a 1 cm optical path. An average of 10 measurements was obtained over five runs, with an equilibrium time of 2 s at 25 °C [45,69]. Considering that xanthan gum solutions are complex systems characterized by concentration-dependent aggregation [70], a Distribution Analysis method was employed. This approach models the correlogram as a summation of intensity contributions from discrete size bands, allowing for a more nuanced resolution of the various molecular species and aggregates present in the presence of other components.

The zeta potential measurements were performed at 25 °C after 2 s of thermal equilibrium time, through the standard Malvern Laser Doppler Velocimetry technique coupled with “M3-PALS phase analysis light scattering”, at a light scattering angle of 173°. The samples were inserted into a polyethylene capillary cuvette coupled to electrodes (DPS1060). The final zeta potential values were calculated as the average of five independent measurements with five counts each, and the data treatment was performed with the aid of the program Microcal Origin 9.0^®^ (OriginLab Corporation, Northampton, MA, USA). Statistical significance was assessed using one-way analysis of variance (ANOVA), followed by Tukey’s multiple comparison test, with a significance level of 0.05.

### 3.4. Rheological Measurements

Rheological experiments were performed to investigate the mechanical response of the samples under different stimuli. The samples were subjected to oscillatory and stationary rheological tests, using a Dynamic Hybrid Rheometer (DHR1; T.A. Instruments^®,^ New Castle, DE, USA), with DIN standard parallel plate geometry (stainless steel, diameter of 40.0 mm) and placed on a Peltier plate for temperature control. Experiments were performed using a gap value of 500 μm and different conditions as described below.

(I) Strain amplitude oscillatory sweeps, from 0.1% to 600% in logarithm sweep mode, collecting 10 points per decade and keeping constant the angular frequency of 1 Hz, were performed at 25 °C in order to determine the region of linear viscoelasticity (LVE) and the possible occurrence of a yield stress (τ_0_).

(II) After the determination of the maximum deformation limit in the viscoelastic moduli (*G*′ and *G*″), the amplitude deformation was fixed at 0.25% (kept inside the LVE) and the oscillation frequency ranged from 0.01 to 100 Hz in logarithmic sweep mode, collecting 10 points per decade, at 25 °C.

(III) Additionally, temperature experiments were performed in oscillatory mode, swept from 5 to 75 °C and from 75 to 5 °C, at 5 °C/min and keeping constant the deformation strain (γ% = 1%) and angular frequency (ω = 1 Hz).

(IV) Steady rheology experiments were also used in order to investigate the behavior of the fluids at high shearing, in a rotational way, to simulate conditions where the microstructures of the fluids are broken by shearing, without time for their reconstitution.

### 3.5. Herbicidal Bioassay

The extract of *S. lappa* is not readily soluble in water, as such, neat dimethyl sulfoxide (DMSO) was used to solubilize it (Figure S3a), and a solution of xanthan gum was used for the dilution of the stock (Figure S3b). Initially, a 0.5% (*m*/*v*) xanthan gum solution was prepared by dissolving 5.00 g in distilled water in a 1.0 L volumetric flask filled to the mark. Subsequently, a 1000 ppm stock solution of *S. lappa* was prepared by first dissolving 600 mg of the extract in 1.8 mL of neat DMSO. This mixture was then diluted to a final volume of 600 mL using the 0.5% gum solution to stabilize it. This procedure resulted in a final DMSO concentration of 0.3% (*v*/*v*) in the stock solution. The 600, 300, 150, and 75 ppm were made using serial dilution from the 1000 ppm stock solution. The negative control (Vehicle Control) consisted of the solvent matrix alone: 0.3% (*v*/*v*) DMSO in 0.5% (*m*/*v*) xanthan gum solution. This control was used to establish the baseline growth and to exclude any potential phytotoxic effects of the solubilizing agents. The commercial herbicide Dual (Dual Gold Syngenta^®^ Company, São Paulo, Brazil; S-metolachlor) was used as a positive control and was prepared at concentrations of 1000, 600, 300, 150, and 75 ppm in DMSO 0.3% (*v*/*v*). The xanthan gum 0.5% (*m*/*v*) solution was not used to prepare positive control solutions.

Clean and dried Petri dishes and their lids were sterilized with 70% ethanol to prevent contamination, and germination papers were placed in them. Twenty seeds of five different plant species—lettuce (*Lactuca sativa*), brachiaria grass (*Brachiaria decumbens*), cucumber (*Cucumis sativus*), beggar tick (*Bidens pilosa*), and sorghum (*Sorghum bicolor*)—were counted into three replicate dishes for each concentration of *S. lappa* extract and Dual Gold. It is well documented in scientific publications that these plant species are sensitive to growth-regulating compounds, which makes them appropriate to be used as bioindicators in herbicidal bioassays [63,66,68,71]. Five milliliters of the corresponding solution were poured into each dish containing the seeds and sealed using a plastic wrap. The dishes were placed in a BOD (Biochemical Oxygen Demand) incubator at 25 °C in the absence of light for five days, after which they were frozen in a freezer for 24 h to halt growth (−18 °C). The frozen dishes were allowed to thaw the next day, and then carefully arranged on 20 cm × 14 cm boards. Digital images of the boards were captured, and the root and shoot lengths of the seedlings were measured using a digital measurer (ImageJ software version 1.54, National Institutes of Health, Bethesda, MD, USA). The RGC of the seedlings in each *S. lappa* solution, as well as in the Dual Gold solution, was computed in relation to the negative control, according to the following Equation (1):

(1) $$RGC(\%)=\frac{S-C}{C}\times 100$$

In this equation, *S* refers to the average length of the germinated shoots or roots of the plants, while *C* denotes the mean growth observed in the negative control (vehicle only). Consequently, the calculated RGC isolates the effect of the extract by normalizing the data against the vehicle’s background activity.

### 3.6. Statistical Analysis

Statistical analyses were performed using Microsoft Excel 365 (Microsoft Corporation, Redmond, WA, USA) and the Statistics Kingdom Web Server (https://www.statskingdom.com). The normality of the data distribution was assessed using the Shapiro–Wilk test [72], while the homogeneity of variance was verified using Levene’s test. Comparisons between treatment groups and the control were conducted using the Kruskal–Wallis test followed by Dunn’s post hoc [73], Welch’s ANOVA followed by Games-Howell post hoc [74], or one-way ANOVA followed by Tukey–Kramer post hoc, depending on the data distribution and variance assumptions. Differences were considered statistically significant at *p* < 0.05.

## 4. Conclusions

In this study, a stable aqueous suspension of the water-insoluble extract of *Saussurea lappa* was successfully formulated using xanthan gum. The physicochemical properties of the suspension showed promising results for its application as agrochemical. According to the results of *D_h_* and *ZP*, at low concentrations, the extract directly interacts with the xanthan gum, leading to smaller and more stable colloidal particles. This microscopic result was also reinforced by the rheology analyses, on a macroscopic level, which demonstrated that the gel’s yield stress (τ_0_) decreases as the concentration of the *S. lappa* increases. The disaggregation of the gum’s network made the suspension weaker and more fluid-like.

The suspension exhibited two desirable properties for use as a commercial sprayable agrochemical. First, the rheological tests confirmed that the solution was a thermally stable weak gel-like viscoelastic system at rest, which prevents sedimentation during storage. Second and most significant, it showed a strong shear-thinning (pseudoplastic) behavior, becoming fluid and easy to spray when high shear is applied, ensuring effective and uniform application when sprayed.

The herbicidal potential of this suspension was evaluated by assessing its impact on the shoot and root growth of five plant species: *Lactuca sativa*, *Brachiaria decumbens*, *Cucumis sativus*, *Bidens pilosa*, and *Sorghum bicolor*. It was observed that the *S. lappa* suspension demonstrated significant and selective phytotoxicity. Its efficacy was comparable to or exceeded that of the commercial herbicide Dual against *L. sativa*, *C. sativus*, and *B. pilosa*. However, the extract exhibited significantly lower efficacy against *B. decumbens* and *S. bicolor*, highlighting its potential as a selective bioherbicide. This result points to potential molecules present in the extract of *S. lappa* that could be used as templates for the synthesis of novel agrochemicals.

The observed herbicidal activity of the *S. lappa* extract could be attributed to the presence of bioactive molecules, such as sesquiterpene lactones (e.g., DHC and costunolide), which have been previously isolated from the plant in other studies [34,62]. Our results obtained with the xanthan gum formulation are consistent with the high-potency herbicidal activity reported in the literature for these isolated compounds and their derivatives. Additionally, our study confirmed that the extract of *S. lappa* with xanthan gum is potentially viable without the need for molecular isolation, which may represent a cheaper and faster approach than the traditional isolation processes.

Furthermore, this work demonstrates that the xanthan gum can be used as an effective, low-cost vehicle for formulating water-insoluble natural products that otherwise could not be utilized as agrochemicals.

## Data Availability

Data is contained within the article and Supplementary Materials.

## Supplementary Materials

The following supporting information can be downloaded at: https://www.mdpi.com/article/10.3390/plants15020337/s1, Figure S1: DHC and its derivatives; Figure S2: Costunolide and its derivatives; Figure S3: The *S. lappa* extract (a) and the xanthan gum-stabilized suspension (b); Figure S4: Analysis of variance (one-way ANOVA followed by Tukey’s multiple comparison test at a significance level of 0.05) for *D_h_* data; Figure S5: Analysis of variance (one-way ANOVA followed by Tukey’s multiple comparison test at a significance level of 0.05) for ZP data; Table S1: Results of bioassays on *Lactuca sativa* (lettuce) seed germination; Table S2: Results of bioassays on *Brachiaria decumbens* (brachiaria grass) seed germination; Table S3: Results of bioassays on *Cucumis sativus* (cucumber) seed germination; Table S4: Results of bioassays on *Bidens pilosa* (beggar tick) seed germination; Table S5: Results of bioassays on *Sorghum bicolor* (sorghum) seed germination.

## Abbreviations

The following abbreviations are used in this manuscript:

BOD	Biochemical Oxygen Demand
DCM	Dichloromethane
DHC	Dehydrocostuslactone
*D_h_*	Hydrodynamic diameter
DLS	Dynamic light scattering
DMSO	Dimethyl sulfoxide
LVE	Linear viscoelasticity
RGC	Relative Growth Change
*ZP*	Zeta potential
