# Supplementary material for: More than a Thickener: Xanthan Gum as a Vehicle for the Herbicidal Extract of Saussurea lappa and Its Rheological Characterization
Source: Plants (Basel). 2026 Jan 22;15(2):337. doi: 10.3390/plants15020337 (PMC12846183; doi:10.3390/plants15020337)
Supplement: Supplementary file 1 [file plants-15-00337-s001.zip › plants-4019966-supplementary.pdf]

SUPPLEMENTARY MATERIAL

More Than a Thickener: Xanthan Gum as a Vehicle for the Herbicidal Extract of Saussurea lappa and its Rheological Characterization

Shafiu Mustapha[1], Bryan N. S. Pinto[2], Ângelo M. L. Denadai[3] and Elson S. Alvarenga[1,*]

1 Department of Chemistry, Universidade Federal de Viçosa, Viçosa 36570-900, MG, Brazil; shafiu.mustapha@ufv.br
2 Instituto Federal de Educação, Ciência e Tecnologia do Espírito Santo, Barra de São Francisco Campus, Barra de São Francisco 29800-000, ES, Brazil; bryan.pinto@ifes.edu.br
3 Pharmacy Department, Universidade Federal de Juiz de Fora, Governador Valadares Campus, Governador Valadares 35032-620, MG, Brazil; angelo.denadai@ufjf.br
* Correspondence: elson@ufv.br

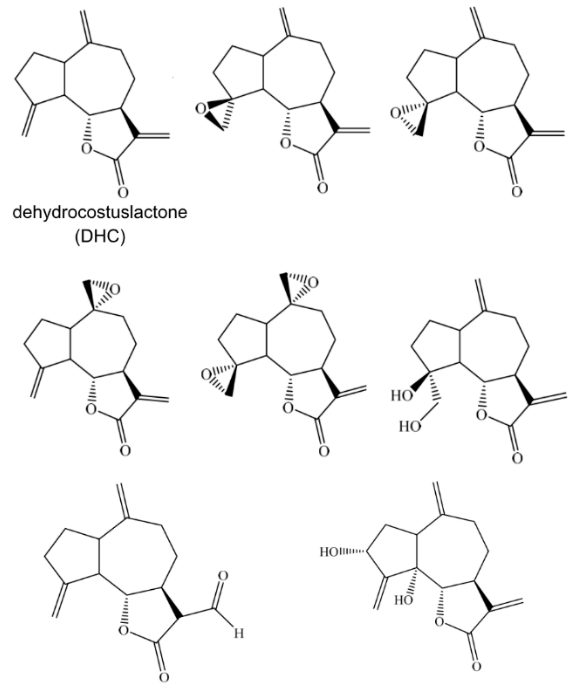

Figure S1 - DHC and its derivatives

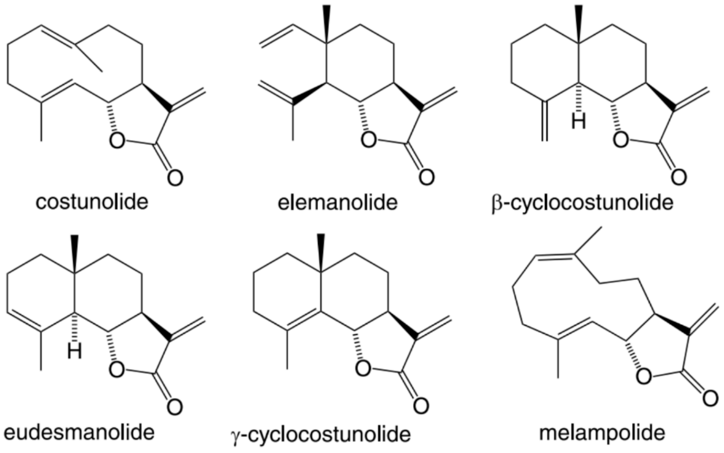

Figure S2 - Costunolide and its derivatives

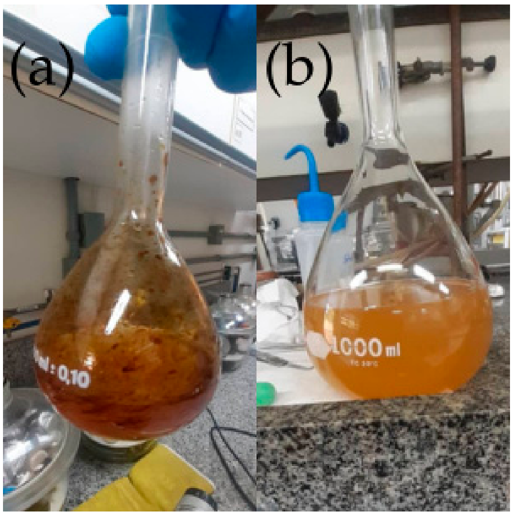

Figure S3 - The *S. lappa* extract (a) and the xanthan gum-stabilized suspension (b)

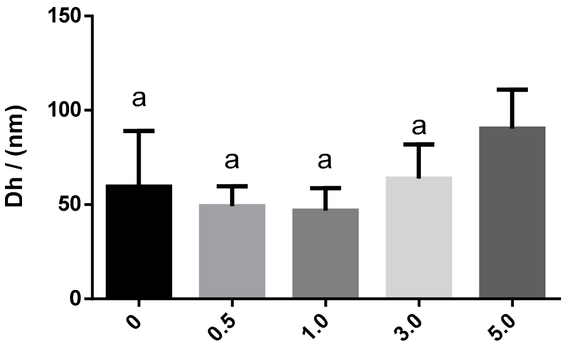

Figure S4 - Analysis of variance (one-way ANOVA followed by Tukey's multiple comparison test at a significance level of 0.05) for D_h data. [a]Statistical difference when compared to Xant 5.0%.

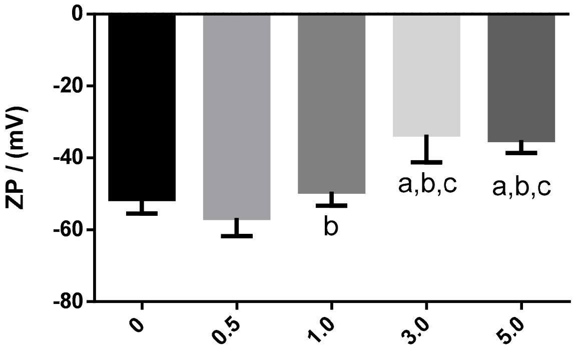

Figure S5 - Analysis of variance (one-way ANOVA followed by Tukey's multiple comparison test at a significance level of 0.05) for D_h data. [b]Statistical difference when compared to Xant 0.5%. [c]Statistical difference when compared to Xant 1.0%.

Table S1. Results of bioassays on *Lactuca sativa* (lettuce) seed germination

	BIOACTIVITY				
	**ROOT**		**SHOOT**		**COMPOUND**
	% from control	S. E.	% from control	S. E.	
75 ppm	-21	11	-75	6	**Reference Compound (Dual)**
150 ppm	-69	8	-86	4	
300 ppm	-56	7	-91	3	
600 ppm	-74	5	-98	1	
1000 ppm	-81	4	-97	1	
75 ppm	30	18	-11	12	***S. lappa* extract**
150 ppm	-26	13	-46	9	
300 ppm	-71	7	-76	5	
600 ppm	-100	0	-100	0	
1000 ppm	-100	0	-100	0	

Table S2. **Results** of bioassays on *Brachiaria decumbens* (brachiaria grass) seed germination

	BIOACTIVITY				
	**ROOT**		**SHOOT**		**COMPOUND**
	% from control	S. E.	% from control	S. E.	
75 ppm	-35	14	-85	4	**Reference Compound (Dual)**
150 ppm	-87	5	-97	1	
300 ppm	-97	2	-99	1	
600 ppm	-97	1	-98	1	
1000 ppm	-100	0	-100	0	
75 ppm	-26	25	-65	13	***S. lappa* extract**
150 ppm	-2	29	-58	14	
300 ppm	-75	10	-95	2	
600 ppm	-87	7	-97	2	
1000 ppm	-100	0	-100	0	

S.E = Standard error

Table S3. Results of bioassays on *Cucumis sativus* (cucumber) seed germination

	BIOACTIVITY				
	**ROOT**		**SHOOT**		**COMPOUND**
	% from control	S. E.	% from control	S. E.	
75 ppm	-69	3	-3	13	**Reference Compound (Dual)**
150 ppm	-87	1	-58	5	
300 ppm	-87	1	-75	5	
600 ppm	-86	2	-89	3	
1000 ppm	-91	1	-96	2	
75 ppm	-31	10	-63	6	***S. lappa* extract**
150 ppm	-19	11	-47	7	
300 ppm	-43	7	-57	6	
600 ppm	-75	4	-68	5	
1000 ppm	-96	1	-93	2	

Table S4. Results of bioassays on *Bidens pilosa* (beggar tick) seed germination

	BIOACTIVITY				
	**ROOT**		**SHOOT**		**COMPOUND**
	% from control	S. E.	% from control	S. E.	
75 ppm	-56	5	-68	5	**Reference Compound (Dual)**
150 ppm	-59	6	-82	3	
300 ppm	-66	5	-82	3	
600 ppm	-75	5	-76	4	
1000 ppm	-87	4	-94	2	
75 ppm	57	50	53	47	***S. lappa* extract**
150 ppm	-36	23	-37	22	
300 ppm	-35	21	-35	21	
600 ppm	-72	12	-76	10	
1000 ppm	-96	3	-96	3	

S.E = Standard error

Table S5. Results of bioassays on *Sorghum bicolor* (sorghum) seed germination

BIOACTIVITY					
	**ROOT**		**SHOOT**		**COMPOUND**
	% from control	S. E.	% from control	S. E.	
75 ppm	-64	6	-65	6	**Reference Compound (Dual)**
150 ppm	-77	4	-76	4	
300 ppm	-87	3	-82	3	
600 ppm	-94	1	-90	2	
1000 ppm	-98	1	-94	2	
75 ppm	23	15	53	19	***S. lappa* extract**
150 ppm	29	16	26	16	
300 ppm	-29	9	-10	11	
600 ppm	-71	5	-22	9	
1000 ppm	-78	3	-51	5	

S.E = Standard error
